# Evidence of carcinogenicity in humans of water-soluble nickel salts

**DOI:** 10.1186/1745-6673-5-7

**Published:** 2010-04-08

**Authors:** Tom K Grimsrud, Aage Andersen

**Affiliations:** 1 Department of Etiological Research, Institute of Population-based cancer research, Cancer Registry of Norway, F Nansens vei 19, Majorstuen, Oslo, Norway

## Abstract

**Background:**

Increased risks of nasal cancer and lung cancer in nickel refiners have been investigated scientifically and discussed since they were detected in the 1930s. Nickel compounds are considered to be the main cause of the cancer excess. Parts of the nickel producing industry and their consultants oppose the classification of water-soluble nickel salts as human carcinogens, and argue that the risk in exposed workers should be ascribed to other occupational exposures and smoking.

**Discussion:**

Respiratory cancer risks in Welsh, Finnish, and Norwegian nickel refiners add to the evidence of carcinogenicity of water-soluble nickel. In Norwegian refiners, the first epidemiological study in 1973 identified high risks of lung cancer and nasal cancer among long-term electrolysis workers. Risk analyses based on exposure estimates developed in the 1980s supported the view that water-soluble nickel compounds were central in the development of cancer. Recently, new exposure estimates were worked out for the same cohort based on personal monitoring of total nickel and chemical determination of four forms of nickel. Additional data have been collected on life-time smoking habits, and on exposure to arsenic, asbestos, sulphuric acid mists, cobalt, and occupational lung carcinogens outside the refinery. After adjustment for these potential confounding exposures in case-control analyses, the risk pattern added to the evidence of an important role of water-soluble nickel compounds as causes of lung cancer. These Norwegian cancer studies rely on national Cancer Registry data, considered close to complete from 1953 onwards; and on National Population Register data continuously updated with mortality and emigration. Canadian mortality studies--perceived to offer the strongest support to the industry position not to recognise carcinogenicity of water-soluble nickel--appear to suffer from limitations in follow-up time, loss to follow-up, absence of risk analysis with individual exposure estimates, no confounder control, and a likely underestimation of cancer mortality.

**Conclusions:**

Rejection to recognise water-soluble nickel as a human carcinogen seems to contradict material epidemiological evidence that demonstrates a strong association between water-soluble nickel compounds and risks of lung cancer and nasal cancer. Independent international scientific bodies have classified nickel compounds as carcinogenic to humans, inclusive of water-soluble nickel.

## Background

In August 2009, the Journal of Occupational Medicine and Toxicology (JOMT) published a paper by Heller *et al. *that questioned the link between water-soluble nickel salts and respiratory cancer [1], followed by an editorial comment [2]. We find it urgent to clarify a number of points in the Heller *et al. *paper, specifically those pertaining to the Norwegian cancer studies in nickel refiners. The nickel and cancer literature covers a period of 70 years, and we offer a description of important parts of the evidence for carcinogenicity in humans of water-soluble nickel.

## Discussion

### A conflict of interest

The editors pointed at a strong conflict of interest for two of the authors of the Heller *et al. *paper (JG Heller and BR Conard) [1,2], who had received funding from Vale Inco; from Xstrata; and from the Nickel Producers Environmental Research Association, NiPERA. For potential readers, it might also be of interest to know that the third author, the late PG Thornhill, worked with the nickel producer Falconbridge Ltd as a metallurgist or director of research for decades until he retired, later serving as a consultant for the company.

Some questions and hypotheses raised by Heller *et al. *[1] have, in fact, been considered and discussed by independent scientists, industry-sponsored consultants, and regulatory boards for 70 years, and hardly deserve to be called 'New views' as suggested in the heading. Some of the ideas can also be found in a recent 53-page review by Goodman *et al.*, and our comments below are partly applicable even to that review [3]. A complete presentation of nickel carcinogenicity would extend beyond the scope of this commentary article, and we will remain focused on statements that we read as questionable claims and possible misconceptions presented in these two industry-funded papers [1,3].

Heller *et al. *[1] unveiled a profound disbelief in some of the central epidemiological studies of respiratory cancer among nickel-refinery workers from the last four decades. They particularly attempt to discredit the contributions from the Cancer Registry of Norway, studies that have been published separately or in combination with international data [4-11]. We appreciate the interest Heller, Goodman and co-workers take in the subject, and admit that we are eager to contribute to the public perception and the scientific community's understanding of the carcinogenic effects of nickel.

Heller and co-workers claimed that water-soluble nickel salts are not carcinogenic, and suggested that the increased lung cancer hazard seen among Norwegian nickel workers exposed to water-soluble forms of nickel should be explained by exposure to insoluble nickel compounds, arsenic, sulphuric acid mists, and smoking [1]. They argue that the epidemiology has failed in recognising these 'true causes' of lung cancer in nickel-refinery workers. These and similar suggestions have been put forward and discussed since the first evidence in the 1970s suggested that water-soluble nickel in the electrolysis might be carcinogenic.

The studies of Canadian nickel workers--from which both Heller *et al. *[1] and Goodman *et al. *[3] seek support--stopped their follow-up of cancer mortality a quarter of a century ago, despite the fact that vital status was unknown at the time for 40 percent of the workers. The authors admitted that they were likely to miss 5 percent of all deaths in the study group. Still, expected mortality numbers were computed as if all workers with unknown vital status were alive throughout the entire observation period, from 1950 through 1984, apparently irrespective of age [12]. This procedure leads to an overestimation of the numbers of expected deaths. The combination of a deficit in the observed numbers and too high expected numbers will result in an underestimation of the observed-to-expected ratio, which is a measure of the relative risk of cancer death compared to the reference population. Heller *et al. *stated that these critical remarks were 'addressed fully' in their Appendix 1 [1], but the appendix contains the same information that was offered by the authors of the original paper 20-25 years ago [12,13].

To our knowledge, there has been no subsequent attempt of improving the data quality for these Canadian nickel workers, and no attempt of assessing the risk according to individually assigned exposure estimates from an exposure matrix, in order to further investigate the role of water-soluble nickel species. Heller *et al. *and Goodman *et al. *remind us that tobacco smoking is an important cause of lung cancer, and that it potentially may confound risk estimates for this disease. We have, however, seen no attempt to adjust for smoking habits in the Canadian studies.

### Respiratory cancer in Norwegian electrolysis workers

The informed reader would recall that the first epidemiological study among Norwegian nickel workers in 1973 demonstrated a high risk of lung cancer and nasal cancer among process workers, both among those with their main experience from roasting and smelting (R/S), and--for lung cancer even more pronounced--among men who had mainly worked in the electrolysis departments [4]. The risks in long-term electrolysis workers were extreme, estimated to 20 and 200 times the expected values for lung cancer and nasal cancer, respectively, compared with the general population.

Qualitative and quantitative differences in nickel exposure between these parts of the refinery indicate a central role for water-soluble forms of nickel. The reasoning is based on the fact that a substantial part of the high total nickel exposures in the R/S departments consisted of insoluble nickel compounds and a correspondingly small proportion was water-soluble salts; whereas the much lower nickel exposures in the electrolysis departments contained mainly water-soluble salts. Thus, the low levels of insoluble nickel species in the electrolysis could not possibly explain the high risks seen in these departments without the existence of another important factor.

Interestingly, even a report from the Clydach refinery, South Wales 15 years earlier pointed at an elevated cancer risk connected with activities that later were recognised to entail substantial exposure to water-soluble nickel: production of copper sulphate and nickel sulphate and cleaning of the underground flues [14]. The production of metal sulphates was performed by separating copper and nickel salts from a mixed aqueous solution by a number of steps involving filtering, precipitation, crystallisation, washing, evaporation, and drying.

Data on cancer incidence among Norwegian refiners rely on compulsory reporting to a national registry of all new cancer cases since 1953. The database of the Cancer Registry of Norway is considered close to complete for the whole population, presently counting about 4.5 million people [15]. For administrative purposes, each inhabitant has a unique personal identity number in the National Population Register, which is continuously updated with deaths and emigrations. The identity numbers effectively secure a high-quality linkage between a given cohort and registry data on vital status, place of residence, mortality, and incident cancers. The cohort of Norwegian refinery workers has been updated and completed with new entrants several times since the first study in 1973 [5-7,9,10], and it has been followed for incident lung cancers through the year 2000 [10].

### Exposure assessment for historical cohorts

The first two Norwegian studies relied on a coarse categorisation of refinery workers into four 'exposure' groups according to each worker's experience at the plant [4,5]. Those who had never been a process worker were grouped separately, while process workers were divided into 'roasting and smelting' (R/S), 'electrolysis', or 'other specified processes', depending on where they had served the longest. This procedure gave approximately equal possibilities to identify cancer risk in the two main groups of process departments, the electrolysis and the R/S departments. The central message from these studies was that water-soluble forms of nickel appeared to be important for the respiratory cancer hazard.

Subsequent studies at the same refinery have tried to specify nickel exposures qualitatively and quantitatively according to department, first based on expert agreement and good knowledge of process chemistry and industrial hygiene [6,7]; and later based on a large number of filter samples and measurements [8]. The studies from the 21^st ^century have all been based on more than 5 thousand measurements of total nickel collected with personally borne sampling pumps in the breathing zone of the most polluted departments between 1973 and 1994. Proportions of water-soluble nickel (and three other forms of nickel) were derived from speciation analyses of dust collected in the 1990s at the same plant, and at other facilities with technology similar to the Norwegian pre-1978 process.

Heller *et al. *[1] and Goodman *et al. *[3] suggested that misclassification in the Norwegian exposure estimates may have created a false picture of a dose-related pattern between water-soluble nickel and cancer risk. Contrary to what Heller *et al. *suggested, the procedure of calculating the year 2000 exposure estimates was no 'revision' of the expert-based estimates from the International study [6]. It is well recognised that retrospective exposure assessment may introduce errors, but estimates based on uniform and presumably unbiased monitoring and measurements through two decades [8] would be expected to have a validity superior to those of an expert judgement limited to four concentration categories of nickel in the air [6]. Additionally, the new quantitative time-department-exposure matrix was developed with contributions from local metallurgists and skilled industrial hygienists [8]. In retrospect, the similarities between the two matrices are more striking than the differences.

Heller *et al. *[1] and Goodman *et al. *[3] suggested that the most recent exposure matrix overestimated the proportions of water-soluble nickel in the roasting and smelting departments, and underestimated the proportion of insoluble nickel species in the electrolysis. The proportions of water-soluble nickel were derived from measurements by the Zatka sequential leaching method [16], which was developed by the nickel producing industry in the early 1990s, and heavily promoted by the same industry for 15 years. As late as 2007, the Zatka speciation method was described by DJ Sivulka, BR Conard, GW Hall, and JH Vincent as 'the most extensively used method to estimate the proportion of various kinds of nickel species in workplace aerosol samples', and it was recommended for the establishment of correct proportions of nickel species for later routine application to measurements of total nickel in the breathing atmosphere [17]. Their paper was sponsored by Inco Ltd and Falconbridge Ltd, and the authors acknowledged the assistance from A Oller at NiPERA. As a recent retreat from this position, the industry has cast doubt on the Zatka method or the way it is used [1,3], but it seems to remain an open question whether any equally applicable method can offer more reliable measurements of the different forms of nickel in workplace exposures.

For the Norwegian cohort, Heller and co-workers proposed that a high turn-over rate and a large number of short-term workers might have added to the observed cancer risks [1]. This suggestion appears to have little relevance, given the repeated demonstration of a risk that strongly increases with length of employment or duration of exposure, especially among electrolysis workers [4,7,10,11]. Furthermore, Heller *et al. *suggested that the observed risks of respiratory cancer might have been boosted by employment of local farmers, who carried with them 'their own acquired risk histories' such as pesticide exposure [1]. By contrast, the Norwegian occupational group of farmers has been shown to carry a significantly lower lung cancer risk compared with national population data, less than half that of the average Norwegian male [18,19].

### Confounder control

We would like to underscore the historical lines in the evaluation of potential confounders. Already in the 1930s--shortly after the recognition of the nickel-refinery hazard--arsenic was mentioned as a possible cause of respiratory cancer [20,21]. Arsenic was virtually eliminated from the Welsh refinery in Clydach during the early 1920s [14], and from the Norwegian refinery in Kristiansand by 1955 [11]. The cancer hazard at each plant, however, has been shown to continue to affect workers with their first entry after these points in time, respectively [10,22]. Moreover, historical exposures to arsenic were adjusted for in the Norwegian analyses in 2005 [11], and the results indicated no more than a minor contribution to the lung cancer incidence.

Potential confounding from smoking has been discussed and addressed repeatedly for the Norwegian cohort since the 1970s [4-7,9-11]. Data on smoking have been included in the studies since 1982 [5]. A study of pulmonary fibrosis conducted among the same workers showed that the correlation was low between individual cumulative exposure to water-soluble nickel and tobacco smoking, expressed as the number of pack-years (Pearson's correlation coefficient = 0.17) [23]. This finding would be in accordance with a weak degree of confounding. For a case-control study of lung cancer, life-time smoking habits were collected from other sources than those of the fibrosis study, and the results showed a strong effect from smoking on lung cancer risk--in line with the expected and commonly seen risks in studies of lung cancer. And, indeed, the confounding effect on the nickel risk estimates, specifically for water-soluble nickel, remained weak (around 15 percent change in the point estimate) [[9], table 8]. This has been explained in an earlier communication [24].

To check for residual confounding from smoking in the case-control study, we included in the regression model continuous variables for total amount smoked, duration of smoking, and years as a former smoker--in addition to the traditional five-category smoking variable reflecting smoking status and intensity--and we found negligible effects on the risk estimates ascribed to nickel [9]. Still, some authors continue to argue: 'Given this lack of data [on smoking among nickel refiners in general, author's comment], one cannot conclude with certainty whether the observed associations between soluble nickel and lung and nasal cancer are real or partially or wholly attributable to smoking' [[3], page 386].

Heller *et al. *hypothesised that exposure to sulphuric acid mists could explain some of the lung cancer risk in nickel refiners [1]. They may have a point in the fact that mists of strong inorganic acids were classified by the International Agency for Research on Cancer (IARC) as a Group 1 human carcinogen in 1992 [25]. But IARC's decision was based mainly on increased risk of larynx cancer in humans. Interestingly, exposures to acid mists were estimated and adjusted for in our 2005 analyses of the Norwegian cohort, and the effect on lung cancer risk was found to be negligible [11].

In experiments reported by Dunnick *et al.*, F344/N rats inhaled nickel sulphate for 2 years and had no increased risk of tumours [26]. A possible explanation to this finding may be limitations in the animal model, such as a low maximum dose obtained in rodents, or species-specific differences in cancer susceptibility for the exposure under study. We therefore find it prudent to warn against the use of animal cancer models in the development of human occupational exposure limits for nickel, as suggested by some authors [27].

### Consistency in the findings

It is interesting to note that the Norwegian epidemiological evidence is relatively consistent over time irrespective of inherent limitations and caveats connected with retrospective exposure assessment. The link between cancer and water-soluble nickel is evident either the risk is estimated according to length of service in departments [4,5,10,11], according to exposure estimates developed by a group of industry experts and independent scientists [6,7], or according to exposure estimates derived from personal monitoring and speciation measurements of nickel from the refinery atmosphere [8-11]. Additionally, there exist epidemiological results from other countries to support the Norwegian findings, from UK (Wales) [6,28], Finland [29], and even from Canada [6], despite the limitations in some of the Canadian cancer data.

We would like to call for a further refining of the historical exposure estimates for the Welsh cohort for the period 1902-1997, and for the Canadian refinery workers, who were exposed between 1914 and 1980--a reassessment that should be performed according to present-day knowledge on speciation, aerosols, and industrial hygiene. Furthermore, we would like to see improvements in the quality of the historical mortality data for Canadian refinery workers, an update of mortality for the last 25 years, and, if possible, to see the reviewed species-specific nickel exposures integrated in risk analyses for the Welsh and Canadian refinery workers.

Interestingly, the nickel producing industry has given priority to this type of research for another cohort which is much less informative, namely the large cohort of U. S. nickel alloy workers [30]. We would like to point at potential shortcomings in the underlying epidemiological study from 1998 [31], of which the most striking limitations seem to be the lack of update of work histories since 1977, a relatively young cohort combined with follow-up through 1988 only, and a possible underestimation of the mortality of the highly relevant disease nasal cancer, due to inconsistent classification of diagnoses.

In the evaluation of carcinogenicity, scientific boards commonly emphasise epidemiological results if available, and review additional evidence from animal experiments and *in vitro *research. The latter has shown great progress during the last decades, and a number of possible mechanisms have been described through which insoluble as well as water-soluble nickel may enhance uncontrolled cell growth and development of cancer--even at low doses and from short-term exposure. The most important modes of action seem to be induction of oxidative stress, inhibition of DNA repair, and other effects operating through epigenetic mechanisms [32,33].

One of the purposes of the International Committee on Nickel Carcinogenesis in Man, which was established in the 1980s, was to put the Norwegian results under scrutiny. After excluding the oldest part of the Norwegian cohort, and focusing on the post-World War II entrants, the results ended up supporting earlier conclusions [6]. Subsequently, several international independent boards have classified 'nickel compounds' as carcinogenic to humans, either directly or indirectly including water-soluble nickel salts. This was done by a working group for IARC in 1990 [34], confirmed in spring 2009 by another IARC working group [35]. Similar decisions were made by the World Health Organisation (WHO) [36], and recently by the European Union's Scientific Committee on Health and Environmental Risks (SCHER) [37].

## Conclusions

We conclude that the industry position not to recognise water-soluble nickel as a human carcinogen appears to contradict material epidemiological evidence and reasonable scientific arguments. Furthermore, consultants to the nickel industry seem to be manufacturing doubt about the ability of water-soluble nickel compounds to cause lung cancer by avoiding to account for a number of epidemiological analyses that have addressed potential confounding factors related to lung cancer risk among exposed workers. Independent international scientific agencies have ruled out chance, confounding, and bias as playing a role in the elevated risk of lung cancer demonstrated in the epidemiological studies, and have classified water-soluble nickel as carcinogenic to humans.

### Addendum

The papers by Heller *et al. *[1] and Goodman *et al. *[3] represent two of a number of publications with funding mainly from the nickel producing industry. Ideally--as stated by the editors of JOMT [2]--scientists involved in research on nickel toxicity should discuss such points as those raised by Heller *et al. *and substantiate the knowledge on nickel toxicity. In cases where this is not done the reader should remember that the economic resources and the scientific commitment in independent research and non-commercial institutions might differ materially from that of large metal producers. In the USA, the reputation of nickel producers has recently been questioned because of their promotion of interests through industry-employed scientists, as described by D Michaels and C Monforton [38]. Recently, one of them, professor David Michaels, who wrote the book 'Doubt is their product' (Oxford University Press), was appointed head of the U. S. Occupational Safety and Health Administration (OSHA).

## Competing interests

The authors declare that they have no competing interests.

The Cancer Registry of Norway is a non-profit institution and did not receive any funding for the preparation of this paper.

## Authors' contributions

The authors contributed equally to this work.
